# The diagnostic value of a nomogram based on multimodal ultrasonography for thyroid-nodule differentiation: A multicenter study

**DOI:** 10.3389/fonc.2022.970758

**Published:** 2022-08-18

**Authors:** Dan Yi, Libin Fan, Jianbo Zhu, Jincao Yao, Chanjuan Peng, Dong Xu

**Affiliations:** ^1^ 1Department of Ultrasound, Shaoxing People’s Hospital, Shaoxing, China; ^2^ Zhejiang Chinese Medical University, Hangzhou, China; ^3^ Department of Surgery, Affiliated Hospital of Shaoxing University, Shaoxing, China; ^4^ Department of Ultrasound in Medicine, Cancer Hospital of the University of Chinese Academy of Sciences (Zhejiang Cancer Hospital), Hangzhou, China; ^5^ Key Laboratory of Head & Neck Cancer Translational Research of Zhejiang Province, Hangzhou, China; ^6^ Zhejiang Provincial Research Center for Cancer Intelligent Diagnosis and Molecular Technology, Hangzhou, China

**Keywords:** contrast-enhanced ultrasonography, elastography, multimodality, thyroid nodules, nomogram

## Abstract

**Objective:**

To establish and verify a nomogram based on multimodal ultrasonography (US) for the assessment of the malignancy risk of thyroid nodules and to explore its value in distinguishing benign from malignant thyroid nodules.

**Methods:**

From September 2020 to December 2021, the data of 447 individuals with thyroid nodules were retrieved from the multicenter database of medical images of the National Health Commission’s Capacity Building and Continuing Education Center, which includes data from more than 20 hospitals. All patients underwent contrast-enhanced US (CEUS) and elastography before surgery or fine needle aspiration. The training set consisted of three hundred datasets from the multicenter database (excluding Zhejiang Cancer Hospital), and the external validation set consisted of 147 datasets from Zhejiang Cancer Hospital. As per the pathological results, the training set was separated into benign and malignant groups. The characteristics of the lesions in the two groups were analyzed and compared using conventional US, CEUS, and elastography score. Using multivariate logistic regression to screen independent predictive risk indicators, then a nomogram for risk assessment of malignant thyroid nodules was created. The diagnostic performance of the nomogram was assessed utilizing calibration curves and receiver operating characteristic (ROC) from the training and validation cohorts. The nomogram and The American College of Radiology Thyroid Imaging, Reporting and Data System were assessed clinically using decision curve analysis (DCA).

**Results:**

Multivariate regression showed that irregular shape, elastography score (≥ 3), lack of ring enhancement, and unclear margin after enhancement were independent predictors of malignancy. During the training (area under the ROC [AUC]: 0.936; 95% confidence interval [CI]: 0.902–0.961) and validation (AUC: 0.902; 95% CI: 0.842–0.945) sets, the multimodal US nomogram with these four variables demonstrated good calibration and discrimination. The DCA results confirmed the good clinical applicability of the multimodal US nomogram for predicting thyroid cancer.

**Conclusions:**

As a preoperative prediction tool, our multimodal US-based nomogram showed good ability to distinguish benign from malignant thyroid nodules.

## Introduction

Thyroid cancer is among the most common endocrine malignancies. In recent years, the detection rate of thyroid nodules has increased due to the popularity of conventional ultrasonography (US), but the incidence of thyroid cancer is only 7–15% (1, 2). As a result, it is necessary to accurately distinguish between benign and malignant thyroid nodules. Although high-frequency US is considered the imaging method of choice (3, 4), it has certain limitations in evaluating benign and malignant thyroid nodules, such as overlapping borders, shape, internal blood flow, and nodule echo, which reduce the diagnostic accuracy for thyroid nodules. As such, benign thyroid nodules may be over-diagnosed and treated. To avoid overtreatment of thyroid lesions, new techniques for improving the thyroid nodule diagnostic accuracy and reducing unnecessary needle biopsy (fine needle aspiration; FNA) and surgery are needed.

Contrast-enhanced US (CEUS) is a relatively recent technology that can reflect and observe the hemodynamics of normal and diseased tissues. It has high sensitivity and specificity and is a valuable auxiliary examination method (5, 6); it has been widely utilized for the evaluation of the liver, kidney, testis, and other parenchymal organs (7). CEUS can also improve the detection rate of blood vessels in thyroid nodules, particularly tiny blood vessels, which is very critical since angiogenesis is the foundation of tumor formation. Therefore, in latest years, CEUS has been increasingly used for thyroid diagnosis in clinical settings, and multiple studies have examined the role of different contrast modes for the diagnosis of thyroid nodules (8, 9). Elastography is a relatively novel technique. By analyzing the deformation degree of normal or sick tissue under the action of external forces, it displays a color map to reflect the hardness of the tissue, which compensates for the shortcomings of the traditional imaging method in terms of obtaining tissue hardness data. It offers a new method for differentiating between benign and malignant nodules (10). Numerous prior research have shown that elastography and CEUS are excellent supplementary techniques for conventional US in identifying benign and malignant thyroid nodules and selecting suitable nodules for FNA (11–13).

A nomogram is a personalized evidence-based graphical method for predicting clinical outcomes. It visualizes complex regression equations, augmenting the readability of predictive-model results and facilitating patient evaluation. Some studies have shown that a nomogram based on The American College of Radiology Thyroid Imaging, Reporting and Data System (ACR TIRADS) classification can effectively improve the ability to identify between benign and malignant thyroid nodules (14). To our knowledge, most of the current studies on thyroid nodules have been single center, and there has been no report on whether a nomogram involving CEUS and elastography features can better discriminate benign from malignant thyroid nodules. Therefore, we attempted to create a multimodal ultrasound-based nomogram for malignancy risk assessment of thyroid nodules, hoping to assist clinical decision making.

## Materials and methods

### Patients

**Figure 1:**
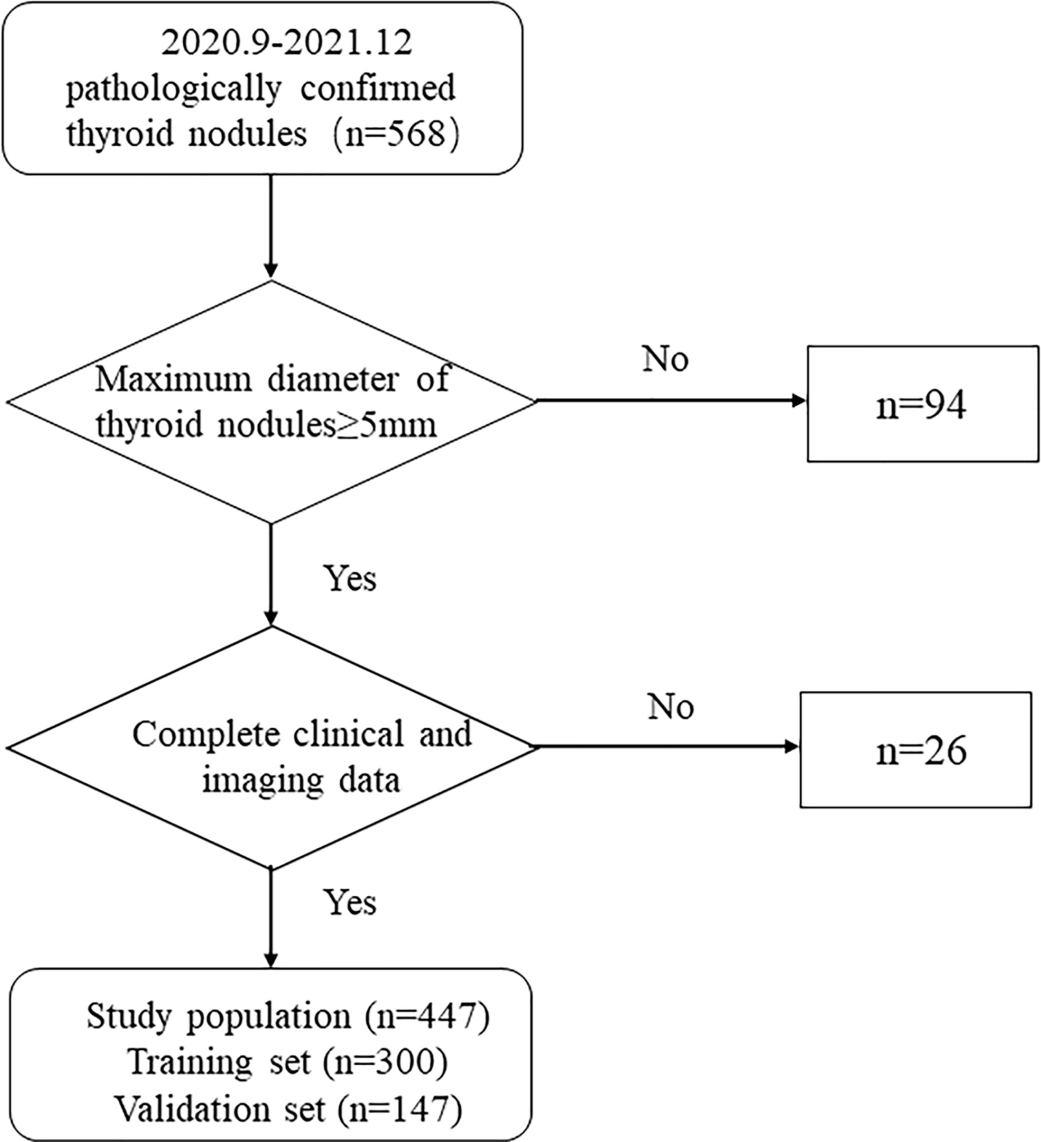
Flowchart of patient selection.

This research consisted 568 individuals with thyroid nodules who met the criteria and whose data were entered in the Medical Image Multicenter Database of the Capacity Building and Continuing Education Center of the National Health Commission from September 2020 to December 2021. The institutional review boards of the numerous participating institutions approved this retrospective study (Clinical Trial ChiCTR2100053599), and the obligation to seek patients’ informed permission was waived. The inclusion criteria were: (a) confirmation *via* surgery or FNA; (b) nodules with a maximum diameter greater than or equal to 5 mm; (c) presence of complete clinical and imaging data. The exclusion criteria were: (a) large nodules without normal parenchyma surrounding as reference; (b) non-repeatedly confirmed Bethesda Class II nodules or changes in size or ACR TI-RADS category found during at least six months of follow-up; (c) allergy to the contrast agent; (d) age younger than 18 years. Finally, 447 patients were included and divided into two sets: 300 patients in the training set (62 male and 238 female, average age:44.61 ± 12.27 years, range: 21–80 years), and 147 patients in the validation set (40 male and 107 female, average age: 46.81 ± 11.93 years, range: 20–75 years). **Figure 1** shows the patient selection flowchart.

Conventional US, elastography, and contrast-enhanced US image analysis were independently analyzed by two experienced sonographers without knowledge of the patients’ clinical information and pathological findings. When disagreements existed, consensus was reached after some debate.

### Conventional thyroid US and ES

The ultrasonic diagnostic equipment Esaote MyLab Class C, GE LogiqE9 and Philips IU22 were employed, and the probe frequency was 5–15 MHz. During the procedure, first, the patient was instructed to remain in the supine position with full exposure of the neck, and the images were independently analyzed and pre-stored by experienced sonographers, who examined the location, size, margin, shape, internal echo, aspect ratio, calcification, vascularity, elasticity score, and other parameters. Longitudinal and transverse images of the thyroid nodules were recorded and saved on the internal hard drive of the instrument for subsequent analysis. All elastography measurements and evaluations followed the recommendations of each machine manufacturer. For example, for the GE machine, following the manufacturer’s recommendation, only cine sequences with 5 green points on the quality bar were used (15). They were classified into three categories based on the echoes of the lesions (16): (a) markedly hypoechoic: echo lower than that of the anterior cervical zonular muscle; (b) hypoechoic: echo between that of the normal thyroid parenchyma and the anterior cervical zonular muscle; (c) iso-echoic or hyperechoic: similar to or higher than the normal thyroid parenchymal echo. The aspect ratio is the ratio of the nodule’s anteroposterior diameter to its left and right diameters (17). We used the Adler blood flow classification to divide it into three grades (18): (a) Grade 0, no blood flow signal within the lesion; (b) Grade I, little blood flow with 1 to 2 punctate or short rod-shaped blood flow signals; (c) Grade II, moderate blood flow with 3 to 4 punctate or one long vessel seen penetrating the lesion with a long diameter approaching or exceeding the radius of the lesion; (d) Grade III, abundant blood flow, with ≥ 5 punctate or two long vessels seen. The internal component of the nodule was divided into solid (characterized as consisting wholly or nearly completely of soft tissue for only a few microscopic cystic interstices), and mixed cystic-solid. When calcification was present, they could be divided into comet tails, microcalcifications, coarse calcifications, and marginal calcifications.

Referring to Asteria et al. (19), the elastography score (ESS) was divided into five modes, ESS 0, red-green, green-blue, blue-red layered distributed in cystic nodules or dominated by cystic nodules; ESS 1, uniformly green (soft); ESS 2, primarily green with little blue dots/areas; ESS 3, primarily blue with little green dots/areas; ESS 4, totally blue (hard).

### Thyroid CEUS

Using the same equipment as that for conventional US, low mechanical index (MI <0.2) was used to perform the examination to limit the destruction of microbubbles and loss of artificial signal. The largest section of the lesion was selected as the CEUS view, showing the entire lesion and surrounding normal glands; the process included switching to the contrast-enhanced ultrasound mode, adjusting the focus so that it was located behind the lesion, keeping the probe still, and instructing the patient to avoid swallowing and talking. According to the instructions, the amount of contrast agent was 2.4 ml per dose, injected into the elbow vein, followed by 5.0 ml of 0.9% saline, and the timer was started while the contrast agent was pushed. The dynamic imaging procedure was observed and acquired for at least 120 seconds, and the ultrasonographic dynamic images were stored.

The qualitative analysis of CEUS included nodule enhanced intensity (no, low, equal, and high enhancement with reference to normal thyroid parenchyma); texture of enhancement (homogeneous, heterogeneous); centripetal enhancement classified as “yes” or “no”; ring enhancement (yes or no), nodule margin after enhancement (clear, unclear); relative washout time divided into fast in, mean time, and slow in; and relative washout timing divided into fast in, mean time, and slow in.

### Intra- and inter-group agreement of ESS and CEUS features

From the study sample, 100 nodules were randomly selected for interobserver and intraobserver reliability investigations of ESS and CEUS features. Data were analyzed independently by two radiologists with similar thyroid experience. One of the radiologists performed a 1-month reassessment of the same nodules to investigate intra-group agreement. The intra-group and inter-group agreement was analyzed using the Cohen kappa coefficient (κ), applying the following range: values of 0.61 to 0.80 were defined as good agreement, and values of 0.81 and above were defined as very good agreement (20).

### Pathology

All pathologies in our research were confirmed *via* FNA or surgery. The final diagnosis was benign when repeat FNA confirmed a Bethesda class II nodule or when no change in nodule size or ACR TIRADS classification was found at least at six months of follow up. All specimens were classified by experienced pathologists who were unaware of the patient’s US findings and medical history.

### Statistical analysis

R software 3.6.3 statistical software (R Foundation for Statistical Computing, Vienna, Austria) and SPSS 25.0 statistical software (IBM Corp., Armonk, NY, USA)were used for analysis. Data that were normally or approximately normally distributed were expressed as mean ± standard deviation using t-tests. Data that were non-normally distributed were expressed as median (interquartile spacing) using Wilcoxon rank sum test. Categorical data were compared using the chi-square or Fisher exact test, expressed as numbers (%). The Kappa consistency test was used to assess intra- and inter-group agreement. Factors that were found to be statistically significant in the univariate analysis were included in the binary logistic regression analysis model, and screened out using the stepwise backward method. Then, according to the multivariate logistic regression analysis results derived using the “rms” package in R, a nomogram for assessing the risk of malignant thyroid nodules based on independent predictive risk factors was constructed. The nomogram assigns a score from 0 to 100 to each variable coefficient. Finally, a total score is read, which predicts the probability of benign and malignant thyroid nodules. The “pROC” package was used to plot receiver operating characteristic (ROC) curves and areas under the curve (AUCs). The rms package was used to plot the calibration curves. To compare the efficacy of the nomogram and ACR TIRADS, decision curve analysis (DCA) was performed using the “rmda” package. P < 0.05 was regarded as statistically significant.

## Results

### Clinical features

**Figure 2:**
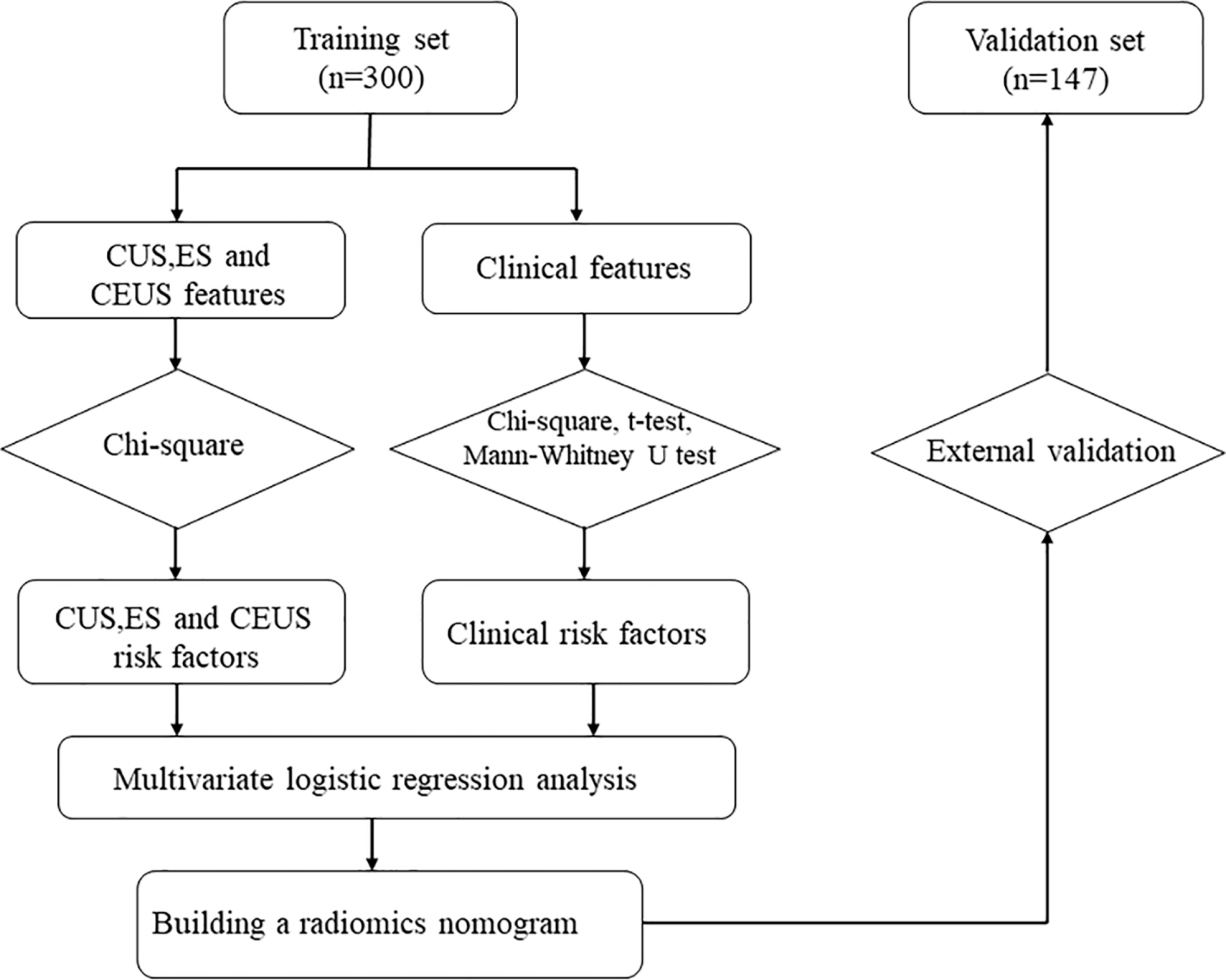
Flowchart of study method CUS, conventional ultrasonography; CEUS, contrast-enhanced ultrasonography; ES, elastography.

**Figure 2** depicts the flowchart for this study. **Table 1** summarizes the clinical characteristics of the two sets. In terms of sex, age, nodule size, location, Hashimoto’s thyroiditis, and pathology, there was no significant difference between the training and validation sets (P > 0.05). The nodule size in the benign group was larger than that in the malignant group in the training set, and the difference was statistically significant (P < 0.001; **Table 2**).

**Table 1:** Clinical features of benign and malignant thyroid nodules.

Clinical features	Training set	Validation set	*P* value
	(n=300)	(n=147)	
Sex			0.121^a^
Female	238 (79.3)	107 (72.8)	
Male	62 (20.7)	40 (27.2)	
Age (years)	44.61 ± 12.27	46.81 ± 11.93	0.073^b^
Hashimoto’s thyroiditis			0.671^a^
Yes	76 (25.3)	40 (27.2)	
No	224 (74.7)	107 (72.8)	
Size (mm)			0.996^c^
Median	10.0	10.0	
Interquartile range	7.0-20.8	6.0-25.0	
Side			0.058^a^
Left	156 (52.0)	64 (43.5)	
Right	138 (46.0)	75 (51.0)	
Isthmus	6 (2.0)	8 (5.5)	
Nodule pathology			0.696^a^
Benign	139 (46.3)	71 (48.3)	
Malignant	161 (53.7)	76 (51.7)	

a: analyzed by the χ^2^ test.

b: analyzed by the t-test.

c: analyzed by the rank sum test.

**Table 2:** Clinical characteristics of benign and malignant thyroid nodules in the training set.

Clinical characteristics	Benign group	Malignant group	*P* value
	(n=139)	(n=161)	
Sex			0.835^a^
Male	28 (20.1)	34 (21.1)	
Female	111 (79.9)	127 (78.9)	
Age (years)	48.02 ± 12.67	41.67 ± 11.14	0.305^b^
Hashimoto’s thyroiditis			0.392^a^
Yes	32 (23.0)	44 (27.3)	
No	107 (77.0)	117 (72.7)	
Size (mm)			<0.001^c^
Median	20.0	8.0	
Interquartile range	10.0-29.0	6.0-11.0	
Side			0.551^a^
Left	69 (49.6)	87 (54.0)	
Right	68 (48.9)	70 (43.5)	
Isthmus	2 (1.5)	4 (2.5)	

a: analyzed by the χ^2^ test.

b: analyzed by the t-test.

c: analyzed by the rank sum test.

### Conventional US, ES, and CEUS characteristics

The conventional US, ES and CEUS characteristics for malignant and benign nodules in the training set are shown in **Tables 3**, **4**. The malignant group had a higher proportion of internal composition (mainly solid), aspect ratio > 1, irregular shape, unclear margin, and microcalcification than the benign group, and the differences were statistically significant (P < 0.05), while the benign group had a higher proportion of internal echo (equal or high) and rich vascularity, and ESS (< 3) than the malignant group, and the differences were statistically significant (P < 0.05). There was no significant difference in internal echo homogeneity between the groups (all, P > 0.05). Compared with the benign group, the enhancement intensity in the malignant group was mostly low (63.1% vs. 23.0%, P < 0.001) with lack of peripheral ring enhancement (95.7% vs. 33.1%, P < 0.001), unclear margin after enhancement (72.0% vs. 23.0%, P < 0.001), and fast-out pattern (23.0% vs. 13.7%, P = 0.039). Benign nodules were more likely to have an echo-free area than malignant nodules (51.9% vs. 12.4%, P < 0.001), showing a fast-in pattern (46.8% vs. 18.0%, P < 0.001). The differences between the two groups in the texture of enhancement and centripetal enhancement were not statistically significant (P > 0.05). Using Cohen’s kappa (κ), we observed good intra- and inter-group agreement for ESS and CEUS features (P < 0.001; **Table 5**). All Cohen’s kappa coefficients were greater than 0.6.

**Table 3:** Conventional ultrasound and elastography (ES) characteristics of benign and malignant thyroid nodules in the training set.

CUS and ES characteristics	Benign group	Malignant group	*P* value
	(n=139)	(n=161)	
Echogenicity			0.026
Hyper-/Isoechoic	8 (5.8)	1 (0.6)	
(Markedly)Hypoechoic	131 (94.2)	160 (99.4)	
Internal composition			<0.001
Solid	87 (62.6)	159 (98.8)	
Mixed (Solid and cystic)	52 (37.4)	2 (1.2)	
Homogeneity			0.734
Homogeneous	10 (7.2)	10 (6.2)	
Heterogeneous	129 (92.8)	151 (93.8)	
Aspect ratio>1			<0.001
Yes	18 (12.9)	85 (52.8)	
No	121 (87.1)	76 (47.2)	
Shape			<0.001
Regular	107 (77.0)	31 (19.3)	
Irregular	32 (23.0)	130 (80.7)	
Margin			<0.001
Well-defined	125 (89.9)	88 (54.7)	
Ill-defined	14 (10.1)	73 (45.3)	
Calcification			<0.001
None or comet-tail artifacts	114 (82.0)	79 (49.1)	
Macro-/peripheral	14 (10.1)	17 (10.5)	
Microcalcifications	11 (7.9)	65 (40.4)	
Vascularity			<0.001
0	15 (10.8)	30 (18.6)	
I	23 (16.5)	73 (45.3)	
II	29 (20.9)	24 (14.9)	
III	72 (51.8)	34 (21.2)	
Elastography score			<0.001
0	5 (3.6)	0 (0.0)	
1	11 (7.9)	1 (0.6)	
2	101 (72.7)	26 (16.2)	
3	18 (12.9)	75 (46.6)	
4	4 (2.9)	59 (36.6)	

**Table 4:** Contrast-enhanced ultrasound (CEUS) features of benign and malignant thyroid nodules in the training set.

CEUS features	Benign group	Malignant group	*P* value
	(n=139)	(n=161)	
Enhanced intensity			<0.001
High	73 (52.5)	17 (10.6)	
Equal	32 (23.0)	44 (27.3)	
Low	32 (23.0)	100 (62.1)	
None	2 (1.5)	0 (0.0)	
Texture of enhancement			0.583
Homogeneous	25 (18.0)	33 (20.5)	
Heterogeneous	114 (82.0)	128 (79.5)	
Echo-free area			<0.001
Yes	75 (51.9)	20 (12.4)	
No	64 (48.1)	141 (87.6)	
Ring enhancement			<0.001
Yes	93 (66.9)	7 (4.3)	
No	46 (33.1)	154 (95.7)	
Wash-in			<0.001
Fast in	65 (46.8)	29 (18.0)	
Slow in/Meantime	74 (53.2)	132 (82.0)	
Wash-out			0.039
Fast out	19 (13.7)	37 (23.0)	
Slow out/Meantime	120 (86.3)	124 (73.0)	
Margin after enhancement			<0.001
Clear	107 (77.0)	45 (28.0)	
Unclear	32 (23.0)	116 (72.0)	
Centripetal enhancement			0.461
Yes	75 (54.0)	80 (49.7)	
No	64 (46.0)	81 (50.3)	

**Table 5:** Intra- and inter-group consistency tests for ESS and CEUS features.

	Intra-group		Inter-group	
	Cohen’s κ (95% CI)	*P* value	Cohen’s κ (95% CI)	*P* value
ESS	0.780 (0.676-0.884)	<0.001	0.810 (0.712-0.908)	<0.001
Enhanced intensity	0.711 (0.593-0.829)	<0.001	0.829 (0.733-0.925)	<0.001
Texture of enhancement	0.640 (0.475-0.805)	<0.001	0.810 (0.685-0.935)	<0.001
Echo-free area	0.844 (0.724-0.963)	<0.001	0.899 (0.801-0.997)	<0.001
Ring enhancement	0.869 (0.757-0.981)	<0.001	0.925 (0.841-1.000)	<0.001
Wash-in	0.742 (0.583-0.901)	<0.001	0.879 (0.763-0.995)	<0.001
Wash-out	0.759 (0.620-0.898)	<0.001	0.865 (0.749-0.981)	<0.001
Margin after enhancement	0.671 (0.526-0.816)	<0.001	0.895 (0.805-0.985)	<0.001
Centripetal enhancement	0.759 (0.632-0.886)	<0.001	0.880 (0.786-0.974)	<0.001

**Figure 3:**
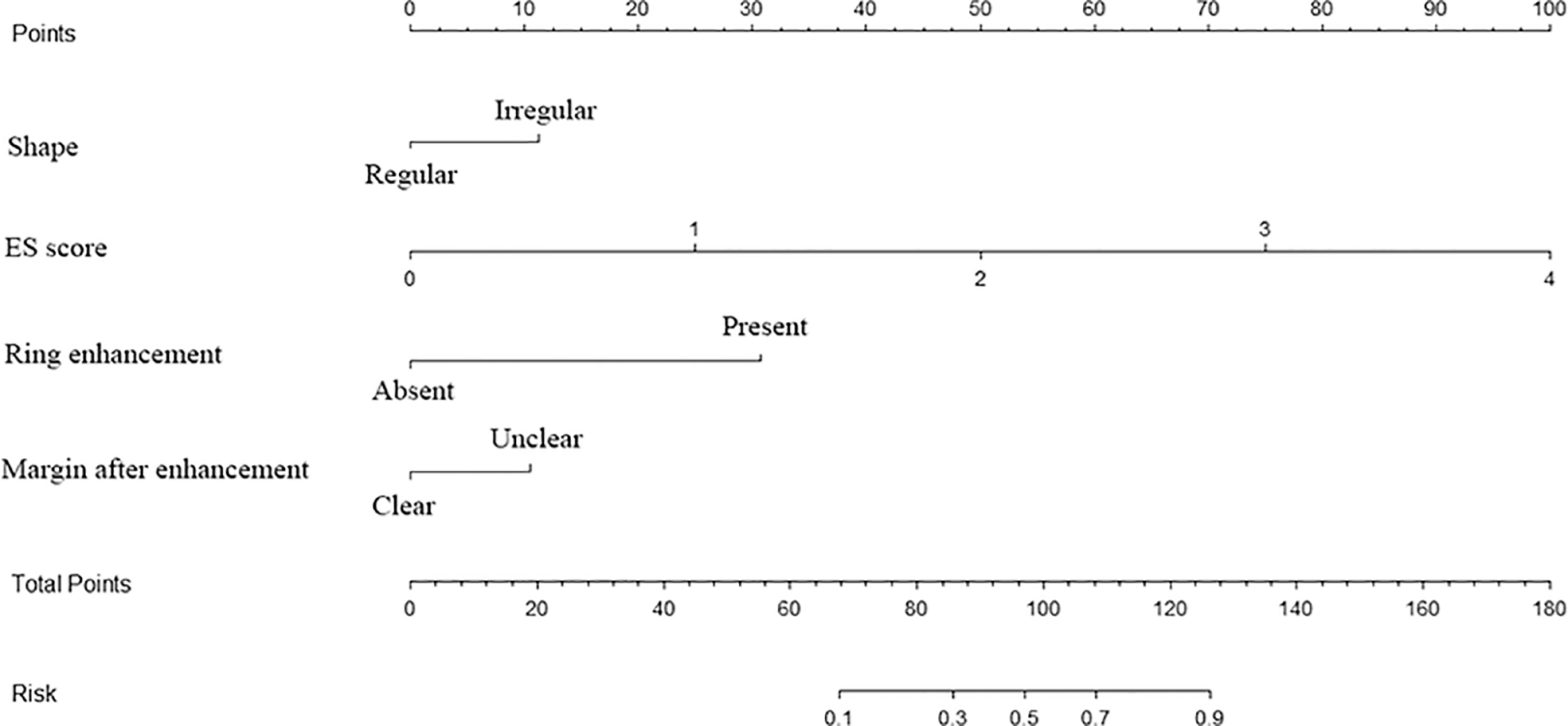
Multimodal ultrasonographic nomogram for predicting risk of thyroid cancer ES, elastography.

**Figure 4:**
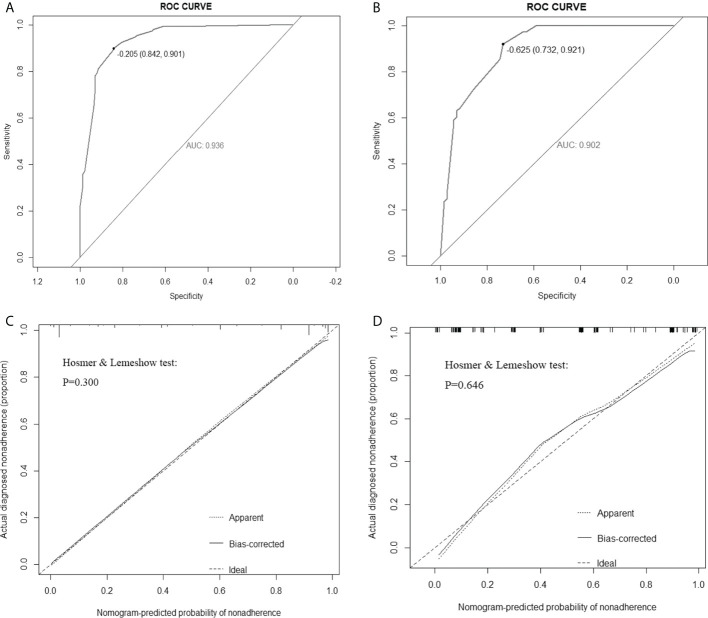
Receiver operating characteristic (ROC) curves of the nomogram in the training set **(A)** and validation set (**B**). Calibration curves of the nomogram in the training **(C)** and validation **(D)** sets. The ideal prediction is represented by the diagonal dashed line, whereas the performance of the nomogram is represented by the solid line. A dashed line closer to the diagonal one suggests a more accurate prediction. AUC: area under the curve.

**Figure 5:**
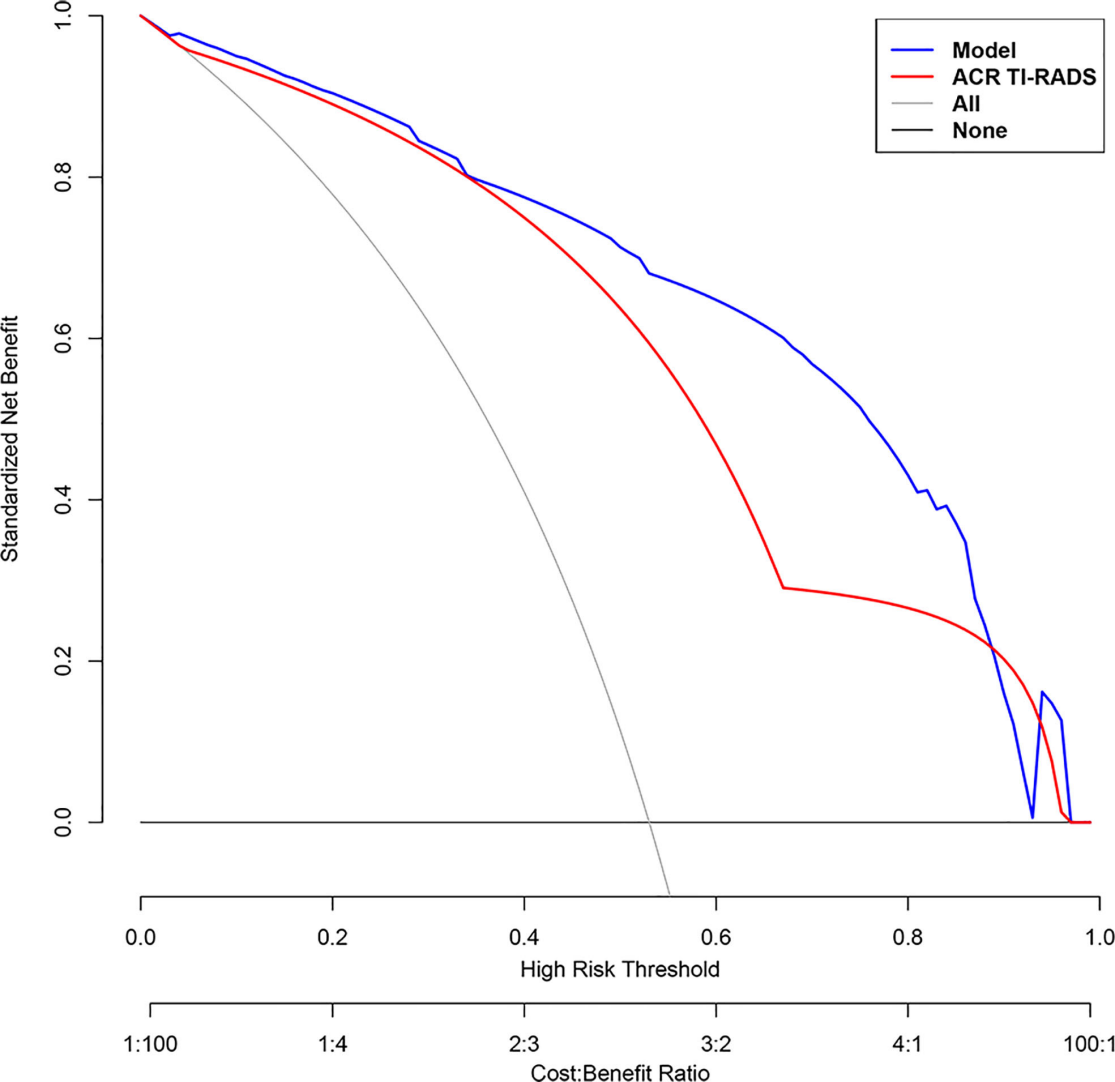
Decision curve analysis (DCA) of each model for predicting the probability of malignancy of thyroid nodules. The standardized net benefit is represented on the vertical axis. The horizontal axis represents the risk threshold. The gray line denotes the presumption that all lesions were cancerous. The DCA results show that the use of the multimodal ultrasonographic nomogram (blue curve) in predicting the probability of malignancy of thyroid nodules thyroid nodules provided greater benefit than using The American College of Radiology Thyroid Imaging, Reporting and Data System (ACR TIRADS) model (red curve) when the threshold probability was between 0 and 0.88.

According to multivariate logistic regression analysis, irregular shape, ESS (≥ 3), lack of ring enhancement, and unclear margin after enhancement were independent predictors of malignant thyroid nodules (P < 0.05; **Table 6**). The ROC curves revealed that the optimal cutoff value for ES was 3. ES had the largest odds ratio (OR) value (OR = 5.395, 95% CI: 3.087–9.429), with specificity, sensitivity, and accuracy of 84.3%, 83.2%, and 86.1%, respectively. According to the above four factors, the malignancy risk nomogram of thyroid nodules was established (**Figure 3**). The ROC curves of the nomogram in the training and validation sets are shown in **Figures 4A, B**. The AUC (95%CI), sensitivity, specificity, and optimal cutoff values of the nomogram in the training and validation cohorts were 0.936 (0.902–0.961), 0.902 (0.842–0.945); 0.901, 0.921; 0.842, 0.732; -0.205, -0.645, respectively (**Table 7**). The calibration curve and Hosmer–Lemeshow test statistic (P = 0.300) of the nomogram are shown in **Figure 4C**. In the training set, the nomogram displayed superior calibration. In the validation set, the good calibration of the nomogram was validated (**Figure 4D**). This demonstrated that the model had an excellent ability to differentiate benign from malignant thyroid nodules. Then, we compared the clinically assessed efficacy of the nomogram with ACR TIRADS in the validation set using the DCA curve (**Figure 5**). DCA showed that using the new model was more beneficial than using ACR TIRADS for clinical diagnosis if the threshold was between 0–0.88.

**Table 6:** Multivariate Logistic regression analysis of benign and malignant thyroid nodules in the training set.

Risk factors	β	*P*	OR	95%CI	
				Lower	Upper
Shape (irregular)	1.104	0.006	3.017	1.374	6.624
Elastography score (≥3)	1.686	<0.001	5.395	3.087	9.429
Ring enhancement (absent)	-2.184	<0.001	0.113	0.038	0.335
Margin after enhancement (unclear)	0.983	0.025	2.672	1.133	6.301
Constant	-5.000	<0.001	0.007	–	–

**Table 7:** Predictive performance of based on internal and external validation.

	Training set	Validation set
Item		
AUC (95%CI)	0.936 (0.902-0.961)	0.902 (0.842-0.945)
Sensitivity	0.901	0.921
Specificity	0.842	0.732
Accuracy	0.870	0.816
Cut-off value	-0.205	-0.645

## Discussion

In our study, we constructed a multimodal US-based nomogram to assess the risk of malignancy in thyroid nodules, which showed good ability to differentiate benign from malignant thyroid nodules. Thyroid cancer is the most common endocrine malignancy in China and other countries (21). Its incidence has increased rapidly worldwide in recent decades. In 2020, the global cancer statistics reported 586,202 new cases of thyroid cancer, 37.7% of which were in China. In China, thyroid cancer has risen to the fourth place among malignant tumors in women, and the incidence is three times that in men (22). Conventional US, as a convenient and cost-effective method for detecting thyroid nodules, has high sensitivity but low specificity (1, 22, 23). Therefore, to avoid overtreatment of thyroid lesions, it is urgent to introduce new technologies to accurately differentiate benign from malignant lesions before performing needle biopsy or surgical resection. 

The ACR TIRADS is a risk stratification system based on US of thyroid nodules published in 2017 by the ACR TIRADS committee (24). ACR TIRADS differs from the American Thyroid Association guidelines, Korean TIRADS, and European Thyroid Association TIRADS in that it uses a point-based risk stratification system and categorizes a greater number of nodules in the “low suspicion, no biopsy” category.

A research by Grani et al. (25) showed the advantage of using ACR TIRADS over other risk stratification systems in that it minimizes the number of needless biopsies of benign nodules by 19.9% to 46.5%. However, according to the findings by Wang et al. (26), the use of ACR TIRADS to estimate the risk of malignancy in thyroid nodules, especially ACR TIRADS category 4 nodules, had low specificity, which may be owing to the overlap between benign and malignant nodules in some features. As a result, ACR TIRADS has some shortcomings in stratifying the risk in thyroid nodules. Additional US technologies, such as elastography and CEUS, are necessary. Recently, the introduction of CEUS has improved the diagnostic ability of conventional US for thyroid nodules. However, there is currently no uniform standard and characteristics for the diagnosis of thyroid malignancies using CEUS, and none of the qualitative or quantitative indicators is sufficiently sensitive (27). Most previous studies were conducted in a single center, and multicenter big data are urgently needed for research and verification.

In this study, we used multicenter data to investigate the effectiveness of a new model combining CEUS and elastography in differentiating malignant from benign thyroid nodules and compared its predictive efficacy with that of ACR TIRADS. Four independent predictive risk factors including irregular shape, ESS (≥ 3 points), lack of ring enhancement, and unclear margin after enhancement were screened out using multivariate logistic regression analysis, and a new model was established based on these four characteristics. We used the new model to detect the malignancy likelihood of thyroid nodules and performed external validation, the AUC of the nomogram in the training and validation cohorts were 0.936 and 0.902.The results of this study indicated that the novel model could predict and observe the probability of thyroid cancer and has good clinical applicability. In our training and validation cohorts, our model outperformed ACR TIRADS.

In most previous studies (13, 28, 29), a contrast pattern of ring enhancement and high enhancement was mostly shown by benign nodules. In contrast, a contrast pattern of low enhancement was mostly shown by malignant thyroid nodules, which is consistent with the findings of this study. This may be attributable to inadequate neovascularization and lack of blood supply in malignant nodules. This study showed that among benign nodules, ring enhancement was seen in 66.9% of cases, and hyperenhancement in 52.5% of instances. Regarding malignant nodules, 62.1% showed low enhancement, whereas 4.3% showed ring enhancement. In the present study, lack of ring enhancement and unclear margin after enhancement were independent predictors of malignant thyroid nodules, possibly because the ring is incomplete or indistinct when the nodule grows unevenly, invading the normal surrounding parenchyma, which perhaps result in lack of ring enhancement or unclear margin after enhancement.

Several previous studies have reported that low lesion elasticity is associated with an increased risk of cancer (30, 31). In many cases, strain-elastic ultrasound has generally outperformed more contemporary techniques, such as shear waves (32, 33). In our research, we used strain elastography The ESS was discovered to be an independent predictor for malignant thyroid nodules in multivariate regression analysis. The OR value of the ESS was the largest (OR = 5.395), with a specificity of 84.2%, sensitivity of 83.2%, and accuracy of 86.1%. That is, the lower the elasticity and the higher the hardness of the diseased tissue, the higher the malignant probability of the nodule, which is consistent with prior findings. Nevertheless, strain elastography is not always feasible in cases where physical compression cannot be applied or where there is lack of normal thyroid tissue that can be compared to nodules. As a result, it is necessary to combine multiple US techniques to increase thyroid nodule diagnostic accuracy.

This present study had several shortcomings. First, it was a retrospective research, and the sample was subject to selection bias; bias and errors tend to be higher in retrospective studies than in prospective studies (34). Second, the analysis of the features of conventional US, ESS, and CEUS was subjective to a certain degree. Third, some nodules were confirmed through cytological reports, and cytological examinations may include false-negative and false-positive results. Finally, although this study used multicenter data, the sample size was not sufficiently large. A larger sample size and prospective study designs are needed for further validation.

In conclusion, we constructed a visual nomogram model to distinguish benign from malignant thyroid nodules based on independent predictive risk factors and validated the model internally and externally. The model was found to have good sensitivity and specificity for predicting thyroid cancer, allowing clinicians and patients to rapidly evaluate the risk of thyroid cancer and reduce unnecessary surgery.

## Data availability statement

The original contributions presented in the study are included in the article/Supplementary Material. Further inquiries can be directed to the corresponding authors.

## Ethics statement

This study was reviewed and approved by Independent Ethics Committee of the Zhejiang Cancer Hospital (IRB-2020-314) and the Chinese clinical trial registry center (ChiCTR2100053599). The Ethics Committees of the other centers that also participated in this study were notified as well. Written informed consent for participation was not required for this study in accordance with the national legislation and the institutional requirements.

## Author contributions

The experimental progress and manuscript writing were handled by DY and LF. The paper was revised by CP and DX. JY was in charge of the experimental design, whereas JZ was in charge of data gathering, analysis, and interpretation. All authors contributed to the article and the final version was approved by all of them.

## Funding

The National Natural Science Foundation of China (no. 82071946) and the Zhejiang Provincial Natural Science Foundation (no.LZY21F030001 and no.LSD19H180001) funded this research.

## Acknowledgments

We would like to thank everyone at the Department of Radiology (ultrasound), Cancer Hospital of the University of Chinese Academy of Sciences (Zhejiang Cancer Hospital) for their invaluable contributions to patient management.

## Conflict of interest

The authors declare that the research was conducted in the absence of any commercial or financial relationships that could be construed as a potential conflict of interest.

## Publisher’s note

All claims expressed in this article are solely those of the authors and do not necessarily represent those of their affiliated organizations, or those of the publisher, the editors and the reviewers. Any product that may be evaluated in this article, or claim that may be made by its manufacturer, is not guaranteed or endorsed by the publisher.
